# RETRACTION: A Meta‐Analysis of the Effect of Different Body Mass Index on Surgical Wound Infection After Colorectal Surgery

**DOI:** 10.1111/iwj.70231

**Published:** 2025-02-11

## Abstract

**RETRACTION**: LiuJ. , LiG. , ChenZ. , and JiangH. , “A Meta‐Analysis of the Effect of Different Body Mass Index on Surgical Wound Infection After Colorectal Surgery,” International Wound Journal 20, no. 6 (2023): 2151–2158, 10.1111/iwj.14091.36860168 PMC10333030

The above article, published online on 01 March 2023, in Wiley Online Library (http://onlinelibrary.wiley.com/), has been retracted by agreement between the journal Editor in Chief, Professor Keith Harding; and John Wiley & Sons Ltd. Following an investigation by the publisher, all parties have concluded that this article was accepted solely on the basis of a compromised peer review process. The editors have therefore decided to retract the article. The authors did not respond to our notice regarding the retraction.

**RETRACTION**: J. Liu , G. Li , Z. Chen , and H. Jiang , “A Meta‐Analysis of the Effect of Different Body Mass Index on Surgical Wound Infection After Colorectal Surgery,” International Wound Journal 20, no. 6 (2023): 2151–2158, 10.1111/iwj.14091.36860168 PMC10333030

The above article, published online on 01 March 2023, in Wiley Online Library (http://onlinelibrary.wiley.com/), has been retracted by agreement between the journal Editor in Chief, Professor Keith Harding; and John Wiley & Sons Ltd. Following an investigation by the publisher, all parties have concluded that this article was accepted solely on the basis of a compromised peer review process. The editors have therefore decided to retract the article. The authors did not respond to our notice regarding the retraction.

